# Can cardiac computed tomography predict cardiovascular events in asymptomatic type-2 diabetics?: results of a long term follow-up

**DOI:** 10.1186/1471-2261-14-2

**Published:** 2014-01-08

**Authors:** Ana Faustino, Rui Providência, Paula Mota, Sérgio Barra, Joana Silva, Andreia Fernandes, Rui Catarino, Susana Basso, Marco Costa, António Leitão-Marques

**Affiliations:** 1 Cardiology Department, Coimbra’s Hospital and University Centre – General Hospital, Coimbra, Portugal; 2 Radiology Department, Coimbra’s Hospital and University Centre – General Hospital, Quinta dos Vales, Martinho do Bispo, Coimbra 3041-801 S, Portugal

**Keywords:** Cardiac computed tomography, Coronary artery calcium, Coronary CT angiography, Cardiovascular risk, Type-2 diabetes, Coronary artery disease

## Abstract

**Background:**

Doubts remain about atherosclerotic disease and risk stratification of asymptomatic type-2 diabetic patients (T2DP). This study aims to evaluate the usefulness of calcium score (CS) and coronary computed tomography (CT) angiography (CTA) to predict fatal and non fatal cardiovascular events (CVEV) in T2DP.

**Methods:**

Eighty-five consecutive T2DP undergoing CT (Phillips Brilliance, 16-slice) with CS and CTA were prospectively enrolled in a transversal case-control study. Patients were followed for 48 months (range 18 - 68) to assess CVEV: cardiovascular death, acute coronary syndrome, revascularisation and stroke. Potential predictors of CVEV were identified. Predictive models based on clinical features, CTA and CS were created and compared.

**Results:**

Performing CT impacted T2DP treatment. Cardiovascular risk was lowered during follow-up but metabolic control remained suboptimal. CVEV occurred in 11.8% T2DP (3.1%/year). CS ≥86.6 was predictor of CVEV over time, with a high negative predictive value, an 80% sensitivity and 74.7% specificity. Although its prognostic value was not independent of the presence/absence of obstructive CAD, adding CS and CTA data to clinical parameters improved the prediction of CVEV: the combined model had the highest AUC (0.888, 95%CI 0.789-0.987, p < 0.001) for the prediction of the study endpoints.

**Conclusions:**

CS showed great value in T2DP risk stratification and its prognostic value was further enhanced by CTA data. Information provided by CT may help predict CVEV in T2DP and potentially improve their outcome.

## Background

Coronary artery disease (CAD) is a leading cause of morbidity and mortality in patients with diabetes mellitus [1,2]. Diabetics have more prevalent, extensive and calcified coronary atherosclerosis than non-diabetics, with an accelerated progression and higher prevalence of multi-vessel disease [3-5]. Type-2 diabetics have also a higher prevalence (26-36%) of silent atherosclerotic lesions and asymptomatic ischemia, making the diagnosis of CAD easier to miss and allowing the disease to progress to an advanced stage before becoming clinically evident [5-10].

Diabetes has been considered a CAD risk equivalent and secondary prevention strategies with antiplatelet therapy and statins have been previously recommended [5,6,11]. However, the Guidelines of the European Society of Cardiology on cardiovascular disease prevention (2012) no longer recommend antiplatelet therapy with aspirin for diabetics without clinical evidence of atherosclerotic disease, due to higher risk of bleeding [12]. There is a wide variation in the risk of cardiovascular events among asymptomatic diabetic patients: while some individuals without coronary plaques are at relative low risk, deriving no benefit from an aggressive therapy, others are high risk individuals who may benefit from more intensive risk modification or even revascularisation [5,12]. Timely detection of silent CAD at an early stage of progression may improve risk stratification of these patients and lead to tailored treatment.

Cardiac computed tomography (CT) has been used to detect CAD at an early stage [6]. Coronary artery calcium score (CS) is a marker of atherosclerosis used to predict the likelihood of significant CAD and myocardial ischaemia, with low radiation exposure and no need of contrast agent. However, it can miss non-calcified CAD [5,13].

Coronary CT angiography (CTA) allows noninvasive visualization of the coronary lumen and wall, detecting both calcified and non-calcified plaque components. It requires contrast agent and exposes patients to higher radiation than CS.

Previous studies have failed to prove the usefulness of CTA or functional tests in screening asymptomatic diabetics [5,7,8,14]. No study to date has demonstrated additional value of CS and CTA when associated to clinical variables and classic risk scores, such as Framingham.

This study aims to assess the additional benefit of CS and CTA, when added to clinical risk stratification schemes, to predict fatal and non fatal cardiovascular events in asymptomatic type-2 diabetics.

## Methods

### Study design

Case-control study enrolling asymptomatic diabetic patients referred for CT from our outpatient clinic. CS and CTA were performed. Clinical and laboratory data were collected from electronic registries concerning both ICD-10 diagnostics and outpatient clinic follow-up. This study was approved by our Institution´s Cardiology Department Supervisor and Ethics Committee. All patients provided informed consent before undergoing CT and authorized the use of follow-up information.

### Patients and eligibility criteria

A total of 85 consecutive type-2 diabetic patients, without history of chest pain or dyspnoea were referred from our hospital’s diabetes outpatient clinic for cardiovascular risk assessment by CT between March 1, 2006, and April 30, 2009. Patients over 18 years old were included in the study. Diabetes was diagnosed according to the American Diabetes Association criteria [15] and patients were on standard anti-diabetic therapy (diet, tablets and/or insulin). Exclusion criteria were any evidence of stroke, carotid disease or peripheral artery disease, other known cardiac diseases, contraindication to iodine-based contrast agents, glomerular filtration rate (GFR) <30mL/min, pregnancy, inability to sustain a 15-second breath-hold, cardiac arrhythmias or uninterpretable CTA.

### Initial data collection

Patients were evaluated during an outpatient visit before undergoing CT. Demographics, clinical data, duration of diabetes, neuropathy, retinopathy, nephropathy, cardiovascular risk factors (hypertension, dyslipidemia, smoking, family history of premature CAD), metabolic syndrome (defined according to ATPIII [16]), body weight, height, waist circumference and blood pressure were evaluated. Laboratorial tests included total cholesterol, triglycerides, high density lipoprotein cholesterol, low density lipoprotein cholesterol, hemoglobin A1c, serum creatinine, C-reactive protein and microalbuminuria. Body mass index, GFR (MDRD formula) and Framingham risk score [17] were calculated. Hypertension and dyslipidemia were defined by a self-reported history or use of specific therapy.

### CT data acquisition

All examinations were performed with a 16-slice CT scanner (Brilliance 16; Philips Medical Systems©, Eindhoven, the Netherlands). A prospective scan without contrast enhancement was performed to measure CS (sequential scan with 8 × 3mm collimation, tube current 55mAs at 120kV, 3mm width), followed by 16-slice contrast-enhanced spiral scan of the heart performed with ECG gating and retrospective post processing. CTA parameters: 16 × 0.75mm collimation, 400ms gantry rotation, pitch of 0.298, tube voltage at 120kV, maximum current of 600—800 mAs depending on patient size, half-scan reconstruction mode and imaging craniocaudal direction. All patients received 5mg of sublingual isosorbide dinitrate 5 minutes before CTA acquisition. Patients with a heart rate >65bpm received 50-200mg of oral metoprolol. A bolus of iodinated contrast agent (370mOsm) was intravenously injected (4-5.5 ml/s). A region of interest was placed in the descending thoracic aorta and image acquisition was automatically initiated using bolus tracking (selected threshold: 110 Hounsfield units [HU]). Images were reconstructed in five phases of the cardiac cycle (0, 37.5, 62.5, 75 and 87.5% of the R-R interval) to minimize motion artifacts. The average radiation dose was 14mSv.

### CT Image interpretation

CT image evaluation was performed on a separate 3D workstation (Brilliance workstation, Philips Medical Systems, Eindhoven, the Netherlands) by two experienced reviewers. CS was measured using the automatic calcium detection algorithm of the workstation, according to Agatston method, with a calcium threshold of 130 HU. CTA were analysed by assessment of axial slices, multiplanar reformations (along the vessel axis and cross-sectional images), and the three thin-slab maximum intensity projections. The coronary artery tree was divided into proximal, medial and distal, according to classic angiographic definition. Plaques were classified as obstructive or non-obstructive using a 50% threshold of luminal narrowing. The presence of obstructive coronary artery disease (CAD: > 50% lumen narrowing) in one vessel (single-vessel disease) or in two or three vessels (multivessel disease) was evaluated. Plaques were defined as structures >1mm^2^ within and/or adjacent to the vessel lumen, distinct from lumen and surrounding tissue. Plaques were classified as: calcified – if they had more than 50% calcified tissue (density >130HU in native scans), mixed – if composed with <50% calcium, and non-calcified lesions - without any calcium. After independent evaluations, the final diagnosis was obtained by a consensus interpretation of the two reviewers.

### Study endpoints

The primary outcome of this study was a combined endpoint of fatal and non-fatal cardiovascular events, including: cardiovascular death (due to cardiovascular causes, obtained according to the death certificate diagnosis), non-fatal acute myocardial infarction (determined from review of hospital case notes and diagnosed according to the Universal Definition of Myocardial Infarction [18]), unstable angina (clinical features of an acute coronary syndrome without diagnostic enzyme changes), revascularisation (excluding that performed immediately after CT), stroke (rapid onset of focal or global neurological deficit lasting ≥24h or leading to death, with clinical findings supplemented by neurological imaging).

### Patient follow-up

Following the CT, patients were followed at the diabetes outpatient clinic according to routine clinical practice. Follow-up was performed between October 1, 2010, and November 30, 2011. Data were obtained by review of clinical and laboratorial records from our hospital’s diabetes outpatient clinic, hospital ward and emergency department admission(s). For patients who were not routinely followed at our institution, an extra follow-up appointment was performed in November 2011.

### Statistical analysis

Statistical analysis was performed using SPSS, version 17.0. Baseline characteristics were described with mean ± standard deviation for continuous data and counts and proportions for categorical data. Continuous variables of time were also described with median, minimum and maximum value. The Kolmogorov-Smirnov test was used to test the normal distribution of continuous variables. The Chi-square test, Student’s t-test and non-parametric equivalent tests were used when appropriate. Regression estimation techniques were applied to replace missing values whenever the number of missing values was negligible, otherwise cases with missing values would be omitted. P values <0.05 (two-sided) were considered statistically significant.

A comparative analysis of diabetics with and without cardiovascular events was performed to evaluate potential predictors. Univariate analysis was performed to evaluate a potential association with the study endpoints. Cox regression (method Forward Conditional) was performed to identify the independent predictors of cardiovascular events over time. Treatment started after CT (revascularisation, antiplatelet agents, statins) and duration of diabetes were also regarded.

The discriminatory power of cardiovascular events predictors was then evaluated through the receiver operating characteristic (ROC) curve, which refers to the ability of a model to assign a higher probability to patients reaching the study endpoint than those who did not reach it. Potential predictors presented as continuous variables were converted into binary variables using as cutoff point the Youden index, which is the point on the ROC curve where optimal sensitivity and specificity are achieved.

Predictor models were created trough multivariate analysis (binary logistic regression with the method Enter) using events predictors (as continuous variables whenever possible): **Clinical model**, comprising GFR, age and Framingham evaluated before CT; **CT model**, comprising CS, obstructive CAD and atherosclerotic plaques; **Clinical-CS model**, including CS and parameters included in Clinical model; and a **Combined model**, composed of parameters included in both Clinical and CT models. The regression coefficients obtained were then applied to calculate predicted risks according to predictor models.

Finally, comparisons of areas under ROC curves (AUC) were performed between predictor models and cardiovascular events predictors using MedCalc for Windows version 9.2.0.1.

## Results

### Study population and CT results

Eighty-five patients were referred for CT. Demographic, clinical and laboratorial characteristics of study population are summarized in Table 1. At study beginning, the median duration of diabetes after diagnosis was 10 years (range 1 - 38 years).

**Table 1:** Study population baseline characteristics

**Patient characteristics**	**n (%)**	**Patient characteristics**	**Mean ± sd**
Male	42 (49.4%)	Age (years)	60 ± 10
Caucasian	85 (100%)	Body mass index (Kg/m^2^)	30.9 ± 4.5
Current smoker	8 (9.4%)	Waist circunference (cm)	104.4 ± 9.2
Ex-smoker	17 (20%)	Duration of diabetes (years)	13 ± 9
Metabolic syndrome	59 (69.4%)	HbA1c (%)	8.2 ± 1.7
Hypertension	79 (92.9%)	Serum creatinine (μmol/L)	69 ± 16.7
Dyslipidemia	68 (80%)	GFR (mL/min/1.73m2)	98.9 ± 25.7
Family history of CAD	8 (9.4)	Urine microalbumin (mg/24h)	61.4 ± 132.4
Insulin treatment	42 (49.4%)	Total cholesterol (mmol/L)	5 ± 1.3
Oral hypoglycaemic therapy	73 (85.9%)	LDL cholesterol (mmol/L)	3.1 ± 1
Statins	45 (52.9%)	HDL cholesterol (mmol/L)	1.2 ± 0.4
ACE inhibitor/ARB	31 (36.1%)	Triglycerides (mmol/L)	1.9 ± 1.3
Antiplatelets	69 (81.2%)	C-reactive protein (mg/dL)	0.38 ± 0.35
Diabetic neuropathy	19 (24.1%)	Framingham	21 ± 12
Diabetic retinopathy/nephopaty	30 (38%)		

Legend: *CAD*, coronary artery disease; *ACE*, angiotensin-converting-enzyme; *ARB*, angiotensin receptor blocker; *GFR*, Glomerular filtration rate.

In our population the median CS was 34 (range 0 - 1293), and a CS > 400 was seen in 9% of the patients. The absence of coronary calcification (CS = 0) was noted in 39% of the patients (Table 2).

**Table 2:** Results of cardiac computed tomography evaluation

**Cardiac computed tomography**	**Results**
**Calcium score, mean ± sd**	**137 ± 250**
Calcium score > 400, n (%)	8 (9.4%)
Calcium score = 0, n (%)	33 (38.8%)
**Coronary angiography**	
**Atherosclerotic plaques, n(%)**	**57 (67.1%)**
Non-calcified, n (%)	12 (14.1%)
Mixed, n (%)	19 (22.4%)
Calcified, n (%)	52 (61.2%)
Calcium score = 0, n (%)	5 (5.8%)
**Obstructive plaques, n (%)**	**21 (24.7%)**
Non-calcified, n (%)	4 (4.7%)
Mixed, n (%)	8 (9.4%)
Calcified, n (%)	10 (11.8%)
Calcium score = 0, n (%)	1 (1.2%)
**Single-vessel disease**	**14 (16.5%)**
**Multivessel disease**	**7 (8.2%)**

By CTA, coronary atherosclerotic plaques were found in 67.1% patients and were obstructive in 23.8%. Five patients (5.8%) with a CS of zero had non-calcified plaques, of which one (1.2%) was obstructive (Table 2). Coronary atherosclerosis was completely absent in 32.9% of patients.

### Follow-up: impact of CT results on treatment and cardiovascular risk profile

Subsequent to CT results, 7.1% of patients underwent percutaneous revascularisation. Medical therapy was optimised in 25.9% patients: 4.7% started ACE inhibitors/ARB´s, 12.9% were put on antiplatelet therapy, and 15.3% were initiated on statins, which was a significant change (p = 0.041). Treatment with an antiplatelet agent, a statin or percutaneous revascularisation was started in 17.6% of patients following CT, which was also a significant change (p = 0.013), and none of these treatments were discontinued. Antidiabetic therapy was intensified in 2.4% of patients (Table 3). Information regarding the cardiovascular risk profile by the time of CT and at time of follow-up is given in Table 4.

**Table 3:** Therapeutic improvement observed after cardiac computed tomography results

**Therapeutic approach**	**Before CT**	**After CT**	**Improvement**	**p**
**Percutaneous revascularisation, n (%)**	0	**6 (7.1%)**	**6 (7.1%)**	**_____**
**Medical therapy, n (%)**			**22 (25.9%)**	**_____**
Antiplatelets	31 (36.1%)	42 (49.4%)	+12.9%	ns
Statins	45 (52.9%)	58 (68.2%)	+15.3%	0.041
ACE inhibitor/ARB	69 (81.2%)	73 (85.9%)	+4.7%	ns
Oral hypoglycaemic agents	73 (85.9%)	74 (87.1%)	+1.2%	ns
Insulin therapy	42 (49.4%)	43 (50.6%)	+1.2%	ns

Legend: *ACE*, angiotensin-converting-enzyme; *ARB*, angiotensin receptor blocker; *p*, significance level.

**Table 4:** Risk factors and risk profile by the time of CT (before) and at time of follow-up (after)

**Risk profile**	**At CT**	**At follow-up**	**p**
**Framingham**, mean ± sd	21.0 ± 11.6	20.8 ± 9.60	ns
**Systolic blood pressure** (mmHg), mean ± sd	158 ± 18	145 ± 21	**<0.001**
**Total cholestero**l (mmol/L), mean ± sd	4.5 ± 1.3	4.4 ± 1.1	ns
**LDL cholesterol** (mmol/L), mean ± sd	2.7 ± 1.1	2.4 ± 0.9	ns
**HDL cholesterol** (mmol/L), mean ± sd	0.7 ± 0.5	0.8 ± 0.4	ns
**Triglycerides** (mmol/L), mean ± sd	1.4 ± 1.3	1.2 ± 0.4	ns
**Hemoglobin A1c** (%), mean ± sd	7.8 ± 1.7	7.2 ± 1.5	**0.049**
**Microalbuminuria** (mg/24h), mean ± sd	61.2 ± 132.4	41.8 ± 97.5	ns
**Glomerular filtration rate** (mL/min/1,73m^2^), mean ± sd	98.4 ± 25.7	80 ± 26.1	**<0.001**
**Waist circunference** (cm), mean ± sd	104 ± 9	106 ± 10	ns
**Current smoking**, n (%)	8 (9.4%)	5 (5.9%)	ns

Legend: p – significance level.

### Follow-up: study endpoints

A median clinical follow-up of 48 months (range 18 - 68) was performed. During this period, 10 cardiovascular events (11.8%) were reported: one unstable angina (1.2%), seven strokes (8.2%) and two cardiovascular deaths (2.4%) – Table 5. No events were observed in patients with zero CS (0% vs. 19.2%, p = 0.007) or without atherosclerotic plaques (0% vs. 17.5%, p = 0.018), both presenting a negative predictive value of 100%. Seven events (in 9.1% of patients) occurred in patients with CS < 400, and three (8.2%) were reported in patients with CS ≥ 400 (Table 5).

**Table 5:** Events occurred during follow-up

**Follow-up: 45 ± 13 months**	**n (%)**
**Cardiovascular events (CVEV)**	10 (11.8%)
**Type of CVEV**	
Acute coronary syndrome	1 (1.2%)
Stroke	7 (8.2%)
Cardiovascular death	2 (2.4%)
**All-cause mortality, n (%)**	3 (3.5%)
Cardiovascular death	2 (2.4%)
Non-cardiovascular death	1 (1.2%)
**CVEV and CS = 0, n (%)**	0 (0%)
**CVEV and CS < 400, (%)**	7 (8.2%)
**CVEV and CS ≥ 400, n (%)**	3 (3.5%)

Length of follow-up was not different between patients with and without cardiovascular events (Table 6).

**Table 6:** Cardiovascular risk factors, markers of diabetes severity, therapeutics and duration of follow-up for patients with (CVEV = 1) and without (CVEV = 0) cardiovascular events

**Univariate analysis**	**CVEV = 1**	**CVEV = 0**	**p**
**Hypertension, CT** (%)	100	92	ns
**Hypercholesterolemia, CT** (%)	80	70.3	ns
**Increased waist circunference, CT** (%)	87.5	84.1	ns
**Metabolic syndrome, CT** (%)	70	69.3	ns
**Duration of diabetes, CT** (years, mean ± sd)	16 ± 8	13 ± 9	ns
**Diabetic neuropathy, CT** (%)	44.4	21.4	ns
**Diabetic retinopathy/nephropathy, CT** (%)	33.3	38.6	ns
**Hemoglobin A1c, CT** (%, mean ± sd)	7.6 ± 1.3	8.3 ± 1.8	ns
**Microalbuminuria, CT** (mg/24h, mean ± sd)	171.9 ± 291.4	45.1 ± 122.6	ns
**Serum C-reactive protein, CT** (mg/dL, mean ± sd)	0.4 ± 0.2	0.4 ± 0.4	ns
**Percutaneous revascularization, CT** (%)	20	5.3	ns
**Antiplatelets, CT** (%)	70	46.7	ns
**Statins, CT** (%)	80	66.7	ns
**ACE inhibitor/ARB, CT** (%)	100	84	ns
**Oral hypoglycaemic therapy, CT** (%)	80	88	ns
**Insulin therapy, CT** (%)	60	49.3	ns
**Hemoglobin A1c, Fup** (%, mean ± sd)	7.7 ± 1.5	7.6 ± 1.6	ns
**Microalbuminuria, Fup** (mg/24h, mean ± sd)	39.3 ± 46.8	39.9 ± 118.4	ns
**Serum C-reactive protein, Fup** (mg/24h, mean ± sd)	0.7 ± 0.4	0.5 ± 0.4	ns
**Antiplatelets, Fup** (%)	66.7	45.8	ns
**Statins, Fup** (%)	55.6	56.2	ns
**ACE inhibitor/ARB, Fup** (%)	77.8	79.2	ns
**Oral hypoglycaemic therapy, Fup** (%)	83.9	86.3	ns
**Insulin therapy, Fup** (%)	66.7	53.4	ns
**Duration of follow-up** (months, mean ± sd)	45.7 ± 15.6	45.2 ± 13.0	ns

Legend: CT – evaluated when computed tomography was performed; for therapeutics, it includes changes performed following CT; Fup – evaluated at time of follow-up; p- significance level; ACE - angiotensin-converting-enzyme; ARB - angiotensin receptor blocker.

On univariate analysis, none of the cardiovascular risk factors, markers of diabetes severity or therapeutics presented in Table 6 were predictors of cardiovascular events. In this analysis, predictors of events were age >66 years old, GFR <99.2mL/min/1.73m^2^ and Framingham >22 when CT was performed, CS > 86.6, atherosclerotic plaques and obstructive CAD on CT, with the primary endpoint occurring in 28.6% patients with vs. 6.3% without obstructive CAD. Treatment with antiplatelet agents, statins and/or revascularisation did not achieve statistical significance. Multivessel disease was not associated with the primary endpoint (Table 7). The cutoff points for age, CS, GFR and Framingham were determined by Youden índex on ROC curve analysis. The best threshold of CS to identify cardiovascular events was 86.6, with 80% sensitivity (vs. 20% for CS ≤ 86.6, p < 0.001) and 74.7% specificity.

**Table 7:** Predictors of cardiovascular events by univariate analysis

**Predictors of CVEV: univariate analysis**	**OR**	**95% CI**	**p**
**Age >66 years**	8.604	1.996 - 37.086	0.001
**GFR <99.2 mL/min/1.73m**^**2**^	9.750	1.176 - 80.829	0.012
**Framingham >22**	4.667	1.111 – 19.602	0.025
**Calcium score >86,6**	11.789	2.299 – 60.445	<0.001
**Atherosclerotic plaques**	1.210	1.076 – 1.367	0.018
**Calcium score ≥400**	6.000	1.177- 30.581	0.018
**Obstructive coronary artery disease**	6.000	1.501 – 23.991	0.018
**Multivessel disease (stenosis)**	------	------	ns
**Female gender**	0.207	0.041-1.042	0.039

Odds ratio (OR) and 95% confidence intervals (CI) and the significance level (p) are shown.

Legend: GFR - Glomerular Filtration Rate.

Presented parameters are related to initial assessment and data obtained by Computed tomography.

**Figure 1:**
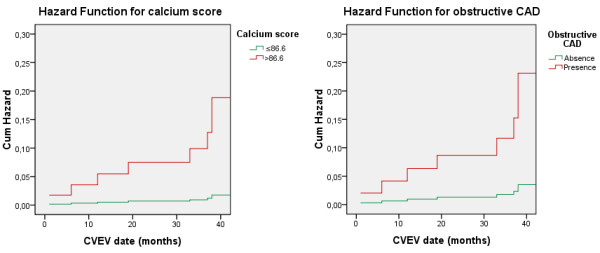
Hazard function by Cox regression showing cardiovascular events along time according to Calcium score higher than 86.6 (vs ≤ 86.6), and the presence of obstructive coronary artery disease (CAD).

All the predictors of cardiovascular events on univariate analysis were included on a Cox regression analysis. GFR (HR 0.953, 95%CI: 0.920-0.988, p = 0.009) and absence of obstructive CAD (HR 0,110, 95% CI: 0.027-0.451, p = 0.002) were independently associated with cardiovascular events over time, behaving as protectors (Table 8). CS > 86.6 was an independent predictor of cardiovascular events over time when considered separately from obstructive CAD (OR 10.725, 95%CI: 2.255-51.018, p = 0.003). Hazard function for primary endpoint along time according to CS higher than 86.6 (vs. ≤86.6) and obstructive CAD is presented in Figure 1.

**Table 8:** Predictors of cardiovascular events by Cox regression analysis

**Predictors of CVEV: Cox regression**	**OR**	**95% CI**	**p**
**Age**	------	------	ns
**Glomerular filtration rate**	0.953	0.920 – 0.988	0.009
**Absence of obstructive coronary artery disease**	0.110	0.027 – 0.451	0.002
**Calcium score**	------	------	ns
**Atherosclerotic plaques**	**------**	------	ns

Presented parameters are related to initial assessment and data obtained by Computed Tomography. Hazard ratio (HR) and 95% confidence intervals (CI) and the significance level (p) are shown.

**Figure 2:**
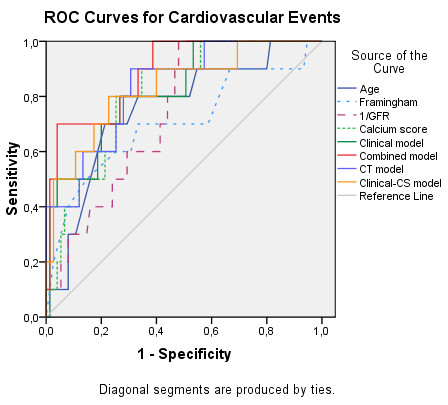
**ROC curve of different parameters and models for identifying patients with cardiovascular events.** Legend: 1/GFR- reciprocal function of glomerular filtration rate (used only for drawing the curve).

Different prediction models were created based on clinical and CT predictors of cardiovascular events. ROC curve analysis for the isolated parameters and the different models is presented in Figure 2 and Table 9. Clinical parameters and calcium score showed good discriminatory power for identifying cardiovascular events. CS had the best AUC of all isolated parameters, while the Combined model, composed of parameters included in both Clinical and CT models, showed the highest discriminatory power among predictor models.

**Table 9:** Receiver operating characteristic (ROC) curve evaluation for CVEV prediction

**Prediction of CVEV: ROC analysis**	**AUC**	**95% CI**	**p**
**Framingham**	0.704	0.506 – 0.902	0.037
**Glomerular filtration rate**	0.262	0.132 – 0.392	0.015
**Age**	0.753	0.596 – 0.911	0.010
**Calcium score**	0.808	0.690 – 0.926	0.002
**Clinical model**	0.823	0.692 – 0.953	0.001
**Clinical-CS model**	0.832	0.692 – 0.972	0.001
**CT model**	0.833	0.711 – 0.955	0.001
**Combined model**	0.888	0.789 – 0.987	<0.001
**ROC comparisons - Combined model vs:**	**AUC (difference)**	**95% CI**	**p**
Glomerular filtration rate	0.150	0.004-0.260	**0.008**
Framingham	0.184	-0.002-0.370	**0.052**
Age	0.135	-0.015-0.284	**0.078**
Calcium score	0.080	-0.022-0.182	0.123
Clinical model	0.065	-0.009-0.140	**0.086**
Clinical-CS model	0.056	-0.023-0.135	0.164
CT model	0.055	-0.025-0.135	0.180

Legend: Predictor models: CT model - composed by calcium score, obstructive Coronary Artery Disease and atherosclerotic plaques; Clinical model – composed by clinical parameters evaluated before Computed Tomography: Glomerular Filtration Rate, age, Framingham; Clinical-CS model – composed by clinical parameters and calcium score; Combined model – composed by parameters included in both clinical and CT models.

Areas under ROC curves (AUC), 95% confidence intervals (CI) and the significance level (p) are presented.

Results presented in this table are related to curves showed in Figure 2.

In assessing cardiovascular events, the Combined model was significantly better than GFR (difference between AUC: 0.150, 95%CI 0.004-0.260, p = 0.008) and showed a trend for a higher discriminatory power than Framingham (difference between AUC: 0.184, p = 0.052), age (difference between AUC: 0.135, p = 0.078 and the Clinical model (difference between AUC: 0.065, p = 0.086). It did not perform significantly better than CS or the other predictor models (Table 9).

## Discussion

Our study suggests that CS can predict cardiovascular events in asymptomatic diabetic patients with a high efficacy and its prognostic power can be further enhanced by CTA results. To our knowledge, no study conducted to date has assessed CTA results (presence of obstructive CAD and atherosclerosis) as predictors of cardiovascular events in asymptomatic diabetics or its additional value over calcium score for cardiovascular events prediction in this population.

In this study, CS was an age-independent predictor of cardiovascular events over time, and its diagnostic performance was better than any isolated clinical parameter (Figure 2, Table 9), cardiovascular risk factor or metabolic control marker (Table 6). The best cutoff for predicting cardiovascular events (86.6) identified 80% of patients who suffered a cardiovascular event and was associated with a risk of events 10.7 times higher over time. It was lower than the value commonly used to predict high risk of CAD in general population (400). A CS of 0, as a low risk marker, missed one patient with significant CAD but did not fail to exclude cardiovascular events, highlighting its negative predictive value.

Evaluation of obstructive CAD and atherosclerosis by CTA increased the CS discriminatory power for cardiovascular events, providing a CT model as accurate as the clinical model. The association of CT and clinical model was more efficient in the prediction of cardiovascular events than GFR, Framingham, age and the Clinical model. Although adding only CS to Clinical model enhanced its discriminatory ability, this difference was not statistically significant for any single parameter, emphasizing the additional value of CTA over clinical model or CS for predicting cardiovascular events.

In asymptomatic subjects from the general population and without known CAD, CS has been shown to predict cardiovascular events above and beyond traditional risk factors [19-23]. However, the predictive value of CS in asymptomatic diabetics is not so well documented. In a large observational study including diabetic and non-diabetic participants, CS predicted all cause mortality in diabetics and added to the predictive power of the Framingham score, while diabetics with undetectable CS had a mortality rate similar to that of non-diabetic individuals [24]. In another study, CS was superior to established risk factors in identifying silent myocardial ischaemia by perfusion scintigraphy in type-2 diabetics [25]. The *PREDICT Study (Coronary calcium measurement improves prediction of cardiovascular events in asymptomatic patients with type 2 diabetes)*[13] also documented CS as a powerful predictor of cardiovascular events in asymptomatic type-2 diabetics that can enhance prediction provided by established risk models. Our data were consistent with those presented in these studies.

Functional tests have also been evaluated to screen asymptomatic diabetics. DIAD (Cardiac Outcomes after Screening for Asymptomatic Coronary Artery Disease in Patients With Type 2 Diabetes) study [14] was a randomised trial that evaluated the impact of ischaemia screening in the prevention of cardiovascular events. Authors concluded that smaller ischaemic defects by adenosine-stress radionuclide myocardial perfusion imaging in asymptomatic diabetic patients were related to a lower event rate. However, the positive predictive value of having moderate or large perfusion defects was only 12%, the total event rate was low (2.9% over a follow-up 0f 4.8 years: 0.6/year) and there were no significant differences between the screened and unscreened group.

Our study population had long diabetes duration and high prevalence of cardiovascular risk factors such as hypertension and dyslipidemia, and metabolic syndrome. Their cardiovascular risk was very high according to Framingham risk score, used for prediction of fatal and non-fatal cardiovascular events, and was reduced over time, due in part to therapeutic improvement performed taking into account CT results.

CTA revealed obstructive CAD in 24.7% of diabetics, a prevalence similar to that reported in previous studies [1,7]. Coronary atherosclerosis was completely absent in 32.9% and this was protective from cardiovascular events, with a negative predictive value of 100%. These results highlight the atherosclerotic heterogeneity of asymptomatic diabetic patients, which was essential to identify and would not be possible without CT.

We found a high event rate (11.8% over 45 ± 13 months, 3.1% per year) in the study population. The addition of CS and CTA data to clinical predictors improved the identification of patients at risk of cardiovascular events, who may benefit from early and potentially more aggressive treatment with statins, antiplatelet agents and revascularisation, as well as tight control of glucose levels.

However, in order to perform CT scan in all asymptomatic diabetics, radiation has to be cut to the minimum. New generation scanners and strategies of dose modulation may significantly reduce radiation exposure to less than 1 mSV.

### Limitations

We describe results of a single-center study, with a limited number of enrolled patients. This was due to a low availability of CT when the study began, but also to the inclusion criteria, as this examination is not routinely performed in asymptomatic individuals. Indeed, our outpatient diabetes clinic receives patients at high cardiovascular risk or with very poor metabolic control, most of which could not be enrolled due to CAD history, ischemic symptoms or low GFR. These data may therefore present a selection bias that may not fit to general asymptomatic diabetic population. A larger sample from other centers would be needed for external validation of these results and of the Combined model created.

Patients´ treatment did not follow a specific protocol, as they were treated according to routine clinical practice of different diabetologists.

We observed therapeutic changes immediately after performing CT, however a control group would be required to evaluate the real impact of CT in cardiovascular events reduction.

## Conclusion

Evaluating atherosclerosis and obstructive CAD through the combination of CS and CTA showed high predictive value for cardiovascular events in asymptomatic type-2 diabetic patients. Furthermore, the use of these two CT methods on top of clinical data improved risk stratification even further, identifying those who can derive the most benefit from intensive prevention measures. The recognition of atherosclerotic disease in this very heterogeneous group of patients led to changes in the therapeutic strategy. However, the true impact of CT risk-stratification and the resulting therapeutic changes on long-term prognosis still needs to be further assessed in randomized controlled trials.

## Abbreviations

CAD: Coronary artery disease; CT: Cardiac computed tomography; CS: Coronary artery calcium score; CTA: Coronary angiography by cardiac computed tomography; CVEV: Fatal and non fatal cardiovascular events; GFR: Glomerular filtration rate; ROC curve: Receiver operating characteristic curve; AUC: Area under receiver operating characteristic curve.

## Competing interests

The authors have no competing interests.

## Authors’ contributions

AF: conception and design, acquisition, analysis and interpretation of data, draft of the manuscript; PM: conception and design, acquisition and interpretation of CT images, analysis and interpretation of data, critical review of the manuscript; JS: conception and design, acquisition of data, critical review of the manuscript; RP: analysis and interpretation of data, critical review of the manuscript; SB: critical review of the manuscript; AFF: acquisition of data; RC: acquisition and interpretation of CT images; SB: acquisition and interpretation of CT images; MC: have given final approval of the version to be published; ALM: have given final approval of the version to be published. All authors read and approved the final manuscript.

## Pre-publication history

The pre-publication history for this paper can be accessed here:

http://www.biomedcentral.com/1471-2261/14/2/prepub
